# Antisense Oligonucleotide-Mediated Silencing of Mitochondrial Fusion and Fission Factors Modulates Mitochondrial Dynamics and Rescues Mitochondrial Dysfunction

**DOI:** 10.1089/nat.2021.0029

**Published:** 2022-01-31

**Authors:** Daniel A. Garcia, Andrew F. Powers, Thomas A. Bell, Shuling Guo, Mariam Aghajan

**Affiliations:** Ionis Pharmaceuticals, Inc., Carlsbad, California, USA.

**Keywords:** antisense, oligonucleotides, mitochondria, mitochondrial dynamics

## Abstract

Mitochondria are highly dynamic organelles that produce ATP and maintain metabolic, catabolic, and redox homeostasis. Mitochondria owe this dynamic nature to their constant fission and fusion—processes that are regulated, in part, by fusion factors (MFN1 and MFN2) and fission factors (DRP1, FIS1, MFF, MIEF1, MIEF2) located on the outer mitochondrial membrane. While mitochondrial fusion and fission are known to influence mitochondrial morphology and function, a key question is whether rebalancing mitochondrial morphology can ameliorate mitochondrial dysfunction in the context of mitochondrial pathology. In this study, we used antisense oligonucleotides (ASOs) to systematically evaluate the effects of fusion and fission factors *in vitro*. Free uptake by cells of fusion or fission factor ASOs caused robust decreases in target gene expression and altered a variety of mitochondrial parameters, including mitochondrial size and respiration, which were dose dependent. In *Mfn1* knockout mouse embryonic fibroblasts (MEFs) and MFN2-R94Q (Charcot-Marie-Tooth Type 2 Disease-associated mutation) MEFs, two cellular models of mitochondrial dysfunction, we found that ASO-mediated silencing of only *Drp1* restored mitochondrial morphology and enhanced mitochondrial respiration. Together, these data demonstrate *in vitro* proof-of-concept for rebalancing mitochondrial morphology to rescue function using ASOs and suggest that ASO-mediated modulation of mitochondrial dynamics may be a viable therapeutic approach to restore mitochondrial homeostasis in diseases driven by mitochondrial dysfunction.

## Introduction

Mitochondria are appreciated for their primary function of energy production to sustain life. While mitochondria have their own genome that encodes 13 mitochondrial proteins, the majority of the mitochondrial proteome is derived from >1,000 nuclear-encoded genes that are synthesized in the cytosol and imported into mitochondria. Among these nuclear-encoded genes are mitochondrial fission and fusion factors that endow these endosymbiotic organelles with their characteristically dynamic nature [1].

Mitochondria exist as a complex interconnected network in the cytoplasm, and their ability to dynamically reshape their morphology is an essential component of cellular homeostasis and adaptive physiology. Mitofusin 1 (MFN1) and mitofusin 2 (MFN2) are GTPases that mediate the outer membrane fusion of neighboring mitochondria, while optic atrophy 1 (OPA1) mediates inner membrane fusion. Reduction of mitochondrial fusion factors results in mitochondrial network fragmentation and has been reported in various contexts [2].

During fission events, the GTPase dynamin-related protein 1 (DRP1) is recruited to the outer mitochondrial membrane where it binds to outer membrane-anchored adaptor proteins (mitochondrial fission 1, FIS1; mitochondrial fission factor, MFF; mitochondrial elongation factor 1, MIEF1 or MID51; mitochondrial elongation factor 2, MIEF2 or MID49) to mediate membrane constriction [2]. FIS1 was originally described as a DRP1 adaptor protein in yeast. However, recent evidence suggests that mammalian FIS1 inhibits the GTPase activity of MFN1, MFN2, and OPA1, thereby preventing fusion rather than activating fission [3]. The function of MFF is clear as a DRP1 adaptor in mammalian cells [4,5], while MIEF1 and MIEF2 have more complex mechanisms of action [6–11]. Nevertheless, reduction of any of the mitochondrial fission factors results in mitochondrial network elongation, and their specific effects on mitochondrial functions are still under investigation.

Fission and fusion are processes critical for maintaining mitochondrial health as mutations in the genes that encode fission and fusion factors cause mitochondrial dysfunction and are associated with various human pathologies. Certain neuropathies, for example, are caused by mutations in *Opa1* (autosomal dominant optic atrophy, ADOA) and *Mfn2* (Charcot-Marie-Tooth disease type 2A, CMT2A), where patient cells have clear mitochondrial morphology imbalance and dysfunction. Abnormal expression of fission/fusion factors and aberrant mitochondrial morphology have also been widely implicated in neurodegenerative, cardiovascular, and metabolic diseases [12].

It is clear that mitochondrial morphology and function are inextricably linked as the structure of the mitochondrial network often dictates a cell's ability to meet energy demands and selectively degrade damaged mitochondria through mitophagy [13]. Thus, in diseases caused by an imbalance of mitochondrial network morphology, a potential therapeutic approach could be to restore normal morphology and function by targeting the opposing fission or fusion pathway to ameliorate disease.

Altering the levels of mitochondrial fission or fusion factors to rebalance mitochondrial morphology has been explored over the past decade. For example, *in vitro* studies in COS7 cells showed that silencing *Drp1*, *Mief1/Mid51*, or *Mief2/Mid49* prevented carbonyl cyanide *m*-chlorophenyl hydrazone (CCCP)-induced mitochondrial fragmentation [6]. In HeLa cells, it was shown that *OPA1* siRNA-induced fragmentation could be rescued by either *Drp1* or *Mff* siRNA, while another study demonstrated that either *Mfn1* or *Mfn2* siRNA reversed mitochondrial enlargement in *Drp1* knockout (KO) cells [4,14]. In mouse embryonic fibroblasts (MEFs), normal mitochondrial morphology was restored in Cofilin KO MEFs using *Drp1* siRNA [15].

Another study used the small molecule leflunomide to rebalance mitochondrial morphology in *Mfn1* and *Mfn2* KO MEFs by inducing *Mfn1/Mfn2* expression, but it was not evaluated whether reduction of fission factors could rebalance mitochondrial morphology and restore mitochondrial function [16]. Furthermore, none of the mitochondrial rebalancing studies reported previously utilized models harboring disease-relevant mutations, limiting the translational potential of the findings.

Antisense oligonucleotides (ASOs) provide a unique opportunity to target mitochondrial fission or fusion to restore the balance of mitochondrial morphology and function that has been disrupted in disease. ASOs affect target gene expression through various mechanisms, including RNase H1-mediated RNA degradation and splicing modulation [17,18], and are routinely used as both tools in research and as therapeutics to affect cellular function and disease [19,20]. In fact, several ASOs have been approved by the FDA to treat a broad range of diseases, and many others are currently being evaluated in the clinic for additional indications [21–26]. The wide use of ASOs in research and the clinic supports the pursuit of antisense therapeutics targeting mitochondrial dynamics.

In this study, we utilize ASOs to potently silence gene expression of each outer mitochondrial membrane fusion and fission factor and systematically evaluate their effects on mitochondrial dynamics and function. We demonstrate that these ASOs modulate mitochondrial morphology, along with changes in mitochondrial respiration and mitophagy. Importantly, we show that ASOs targeting *Drp1* can rescue mitochondrial morphology and respiration by reducing mitochondrial fission in cells that exhibit excess mitochondrial fragmentation and dysfunction, including an *in vitro* disease model of CMT2A. This study shows for the first time that ASOs targeting mitochondrial fission factor *Drp1* can bring balance to mitochondrial network morphology and restore respiration in cellular models of mitochondrial dysfunction.

## Materials and Methods

### Synthesis of ASOs

All ASOs are 16 nucleotides in length and chemically modified with a phosphorothioate backbone and 2′-4′ constrained ethyl (cEt) in the three nucleotides at each end (Supplementary Fig. S1). ASOs were synthesized at Ionis Pharmaceuticals, as previously described [27]. In brief, ASOs were synthesized at 40 μmol scale using UnyLinker™ solid support. cEt bridged nucleic acid (BNA) phosphoramidites were synthesized using reported procedures [27]. A measure of 0.1 M solution of cEt BNA in 50% toluene in acetonitrile and standard sulfur transfer, detritylation, and capping reagents were used for ASO synthesis. For each of the modified analogs fourfold excess of modified nucleoside 3′-phosphoramidite was delivered with a 12-min coupling time.

Postsynthetically, all oligonucleotides were treated with 1:1 triethylamine:acetonitrile to remove cyanoethyl protecting groups from the backbone phosphorothioate linkages. Subsequently, solid support bearing ASOs were treated with aqueous NH_4_OH (28–30 wt%) and heated at 55°C for 18 h. The solid support was then filtered and washed with water. Filtrate and washings were pooled together and purified by high-performance liquid chromatography (HPLC) on a strong anion exchange column (GE Healthcare Life Sciences SOURCE 30Q) using a linear gradient (buffer A: 100 mM NH_4_OAc in water containing 30% acetonitrile, buffer B: 1.5 M NaBr in buffer A, flow 4 or 10 mL min^−1^, *λ* 260 nm). Fractions containing full length ASO were pooled together, concentrated, and desalted by HPLC on a reverse phase column. Purity and mass of oligonucleotides were determined using ion-pair liquid chromatography–mass spectrometry analysis. ASOs were formulated in PBS, sterile filtered, and added to cell culture media. For simultaneous administration of two ASOs, 5 μM of each ASO were used.

### Cell culture and reagents

All cell cultures were maintained at 37°C with 5% CO_2_. mouse hepatocellular SV40 large T-antigen carcinoma (MHT) cell line (described previously, [28]), WT MEFs (ATCC), *Mfn1* KO MEFs (ATCC), *Mfn2* KO MEFs (ATCC), MFN2-R94Q MEFs, and Lenti-X-293T (Clontech) cell lines were cultured in DMEM with 10% FBS. All cell lines tested negative for mycoplasma contamination (IDEXX BioAnalytics). Lentiviral particles were produced using the Lenti-X-293T cells and Lenti-X Single Shots system (Clontech) and used to transduce MHT and MEF cell lines. Cells with stable expression of vectors were selected with 1 μg/mL puromycin. MFN2-R94Q lentiviral vectors were created by performing site-directed mutagenesis (QuikChange II Kit; Agilent Technologies) on a mouse *Mfn2* tagged TrueORF clone (Origene) and then cloned into lentiviral gene expression vector pLenti-C-Myc-DDK-P2A-Puro (Origene).

Basal mitophagy was quantified using the Mito-QC mitophagy reporter [29]. Briefly, MHT cells were transduced with pLVX-mCherry-GFP-FIS1 (MRC PPU Reagents No. DU55501) lentiviral particles, and stably expressing clones were selected with 1 μg/mL puromycin.

### Western blot analysis

Whole cell lysates were prepared from cultured cells using RIPA buffer (Thermo Fisher Scientific) and cOmplete ULTRA Protease Inhibitor Cocktail tablets (Millipore-Sigma). All lysates were sonicated then clarified by centrifugation at 10,000 rpm for 10 min at 4°C. Total protein in supernatants was quantified using the DC Protein Assay (Bio-Rad). Thirty micrograms of protein from each sample was denatured with 4 × NuPAGE LDS Sample Buffer (Thermo Fisher Scientific) supplemented with 8% β-mercaptoethanol, separated on NuPAGE 4%–12% Bis-Tris SDS-PAGE gels with 1 × MES running buffer (Thermo Fisher Scientific), and transferred to nitrocellulose membranes (Bio-Rad). Membranes were blocked with 5% nonfat milk (Bio-Rad) in 1 × PBS supplemented with 0.02% Tween-20 (PBST) at room temperature for 1 h and probed with primary antibodies in diluted blocking solution overnight at 4°C.

Primary antibody signals were detected using fluorescent secondary antibodies (LI-COR) and imaged using the Odyssey instrument (LI-COR Biosciences). Western blot band densities were quantified using ImageJ, and each sample was normalized using beta-actin (ACTB) as a loading control. Values are reported relative to PBS- or Control ASO-treated samples. Primary and secondary antibodies used can be found in the Supplementary Data.

### Reverse transcription quantitative real-time PCR

Total RNA was prepared from cells grown in 96-well plates by first lysing cells in Buffer RLT (Qiagen) supplemented with 8% β-mercaptoethanol (Sigma-Aldrich). RNA was then isolated using an RNeasy 96 Kit (Qiagen). For each biological replicate, reverse transcription quantitative real-time PCR (RT-qPCR) was performed in technical duplicates with 96-well plates containing RNA, EXPRESS One-Step SuperScript Kit (Thermo Fisher Scientific), and 10 μM TaqMan primer probe sets in 20 μL of total volume per well. StepOnePlus real-time PCR instruments (Applied Biosystems) were used to quantify relative gene expression using the ΔΔCt method against a standard curve. The following thermocycling protocol was used at 50°C for 15 min, 95°C for 2 min and then 40 cycles of 95°C for 15 s and 60°C for 1 min. Relative gene expression was normalized by *Cyclophilin A* (*Ppia*) reference gene expression. Primer and probe sequences can be found in the Supplementary Data.

### Mitochondrial DNA analysis

Total DNA was isolated from cells using a DNeasy 96 Kit (Qiagen). For each biological replicate, quantitative PCR was performed as described above, excluding the SuperScript Mix reagent in the RT-qPCR Kit and using the following thermocycling protocol (50°C for 2 min, 95°C for 10 min, then 40 cycles of 95°C for 15 s and 60°C for 1 min). Relative mitochondrial DNA (mt-DNA) amount was determined using a mt-DNA-specific primer probe set (*mt-Nd2*) and normalized by a reference gene of nuclear origin (*Gusb*). Primer and probe information can be found in the Supplementary Data.

### Immunofluorescent microscopy and image analysis

For mitochondrial morphology studies, cells were cultured in chambered coverslip slides (Ibidi), treated with ASOs, and then stained with 200 nM MitoTracker Red CMX-Ros (Thermo Fisher Scientific) for 30 min at 37°C. Cells were then fixed in 4% paraformaldehyde diluted in culture media for 15 min at 37°C and stained with Hoechst 33342 solution (Thermo Fisher Scientific) diluted 1:1,000 in 1 × PBS to detect nuclei. Confocal imaging was conducted on fixed cells, and 30-step Z-stacks were acquired (Leica SP8, 63X oil objective). Images were processed and analyzed using the Imaris software package (Bitplane). Three-dimensional (3D) models of the MitoTracker signal were constructed using the Imaris Surfaces Module, and mitochondrial length was quantified using the Imaris Filament Tracer module.

For mitophagy quantification, cells stably expressing the lentiviral mCherry-GFP-FIS1 mitophagy reporter vector were cultured in chambered coverslip slides (Ibidi), treated with ASOs, and imaged live (Leica SP8, 63X oil objective). Thirty-step Z-stacks were acquired and then processed and analyzed with Imaris to create 3D models of the mitophagy reporter signal. To quantify total normal mitochondria, a 3D model of the GFP signal was used to determine total mitochondrial volume. To quantify mitophagy events, the GFP signal was subtracted from the mCherry signal to isolate mitophagy events (mCherry signal alone), and a 3D model was created from this resulting signal. Mitophagy index was calculated per image and is defined as the ratio of the volume of mCherry signal alone (mitophagy) to the volume of GFP signal (total mitochondria). For all microscopy experiments, *n* = 4–9 images per group, and each image contained 3–6 cells.

### Mitochondrial respiration analysis

Mitochondrial function was assessed using the XFe24 Seahorse Analyzer (Agilent Technologies). Seahorse plates (Agilent Technologies) were coated with 50 μL of Cell-Tak (22.4 μg/mL; Corning) in 0.1 M sodium bicarbonate buffer (supplemented with NaOH according to manufacturer's instructions) for 20 min at room temperature. After washing the plates twice with 500 μL H_2_O and air dried, cells treated with ASOs for 48 h prior were plated at 50,000 per well in 100 μL of Assay Media (Seahorse XF DMEM supplemented with 10 mM glucose, 1 mM pyruvate, and 2 mM glutamine). Cells were adhered to the well surface by centrifuging for 1 min at 200*g* (no brake). Loaded plates were then equilibrated at 37°C in an incubator without CO_2_ for at least 30 min. Following equilibration, 400 μL of Assay Media was added to each well and incubated for an additional 30 min.

Mito Stress Tests (Agilent Technologies) were performed according to the manufacturer's instructions. Briefly, Seahorse XFe24 Sensor Cartridges were hydrated with Seahorse XF Calibrant solution overnight at 37°C (no CO_2_) the day before performing the assay. Cells were stimulated sequentially with one injection each of 1 μM oligomycin, 2 μM trifluoromethoxy carbonylcyanide phenylhydrazone (FCCP), and 0.5 μM rotenone/antimycin A. Mix, wait, and measure times were 3 min each. Three measurements were made following each injection. Oxygen consumption rates (OCRs) were normalized by relative cell number using CyQUANT Direct (Thermo Fisher Scientific) fluorescence intensity measured with a Spark microplate reader (Tecan). Data were analyzed with Wave software (Agilent) and exported to Prism (GraphPad).

### Flow cytometry analysis

Cells were cultured and treated with ASOs as described and then stained with 20 nM MitoTracker Green (Thermo Fisher Scientific) or 50 nM tetramethylrhodamine ethyl ester (TMRE) (Millipore-Sigma) for 30 min at 37°C. Trypsinized cells were resuspended in PBS supplemented with 1% FBS and analyzed using the Accuri C6 instrument (BD Biosciences). Cell debris was excluded, and live cells were gated based on forward scatter versus side scatter dot plots. Live cells were plotted on histograms, and mean fluorescence intensity was determined using FlowJo software. Unstained cells were used as negative controls, and CCCP treated cells were used as a positive control for TMRE staining (data not shown).

### Statistical analyses

Data are reported as mean or median ± SEM. Statistical analyses were performed with an unpaired *t*-test, one-way ANOVA, or two-way ANOVA with multiple comparisons using Prism (GraphPad). Appropriate statistical tests and sample sizes are indicated in figure legends.

## Results

### ASOs targeting mitochondrial fusion and fission factors exhibit potent gene silencing and alter mitochondrial morphology

**FIG. 1.**
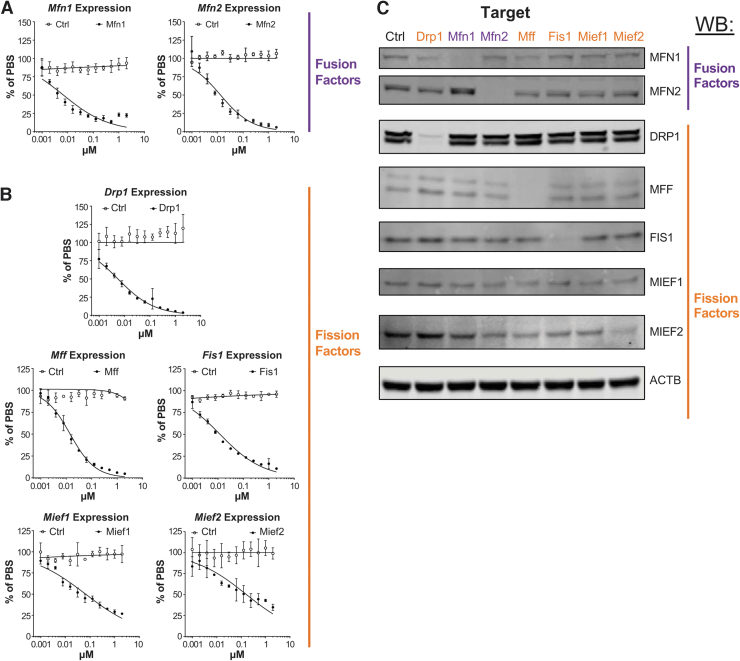
ASOs targeting mitochondrial fission and fusion factors exhibit potent gene silencing *in vitro*. Dose-responsive activity of ASOs targeting mitochondrial fusion factors **(A)** and fission factors **(B)** measured using RT-qPCR of mRNA from MHT cells following 48 h of ASO treatment. *Open circles* represent Control ASO, and *filled circles* represent indicated ASO. Gene expression levels reported are mean (±SEM, *n* = 3) relative to cells treated with ASO vehicle (% of PBS). IC_50_ values for each ASO are 6.4 nM (Mfn1), 12.8 nM (Mfn2), 5.6 nM (Drp1), 14.2 nM (Mff), 11.9 nM (Fis1), 65.7 nM (Mief1), and 160 nM (Mief2) and were determined by performing nonlinear regressions with least squares fit in Prism (GraphPad). **(C)** Western blots of whole cell lysates from MHT cells treated with 5 μM of indicated ASO for 48 h. Primary antibodies used to detect protein levels are indicated. Beta-actin (ACTB) served as a loading control. Western blot quantification is shown in Supplementary Fig. 2C. ASOs, antisense oligonucleotides; MHT, mouse hepatocellular SV40 large T-antigen carcinoma cell line; RT-qPCR, reverse transcription quantitative real-time PCR.

To determine whether ASOs can be used to modulate mitochondrial dynamics, we administered a panel of ASOs targeting mitochondrial fusion and fission factors (*Mfn1*, *Mfn2*, *Drp1*, *Fis1*, *Mff*, *Mief1*, *Mief2*) to MHT (mouse hepatocellular carcinoma) cells in culture [28]. MHT cells freely internalize ASOs in media, and after 48 h of treatment, we found that all ASOs potently reduce their target mRNA in a dose-responsive manner, with IC_50_ values ranging from 5.6 to 160 nM (Fig. 1A, B). ASO treatment also resulted in corresponding decreases in target protein levels (Fig. 1C). Treatment with a nontargeting control ASO did not alter the expression of any target we measured, and while some modest compensatory changes in protein expression of other fusion/fission factors were observed following target ASO treatment, factor levels remained generally unchanged across all conditions (Fig. 1C and Supplementary Fig. S2A–C).

**FIG. 2.**
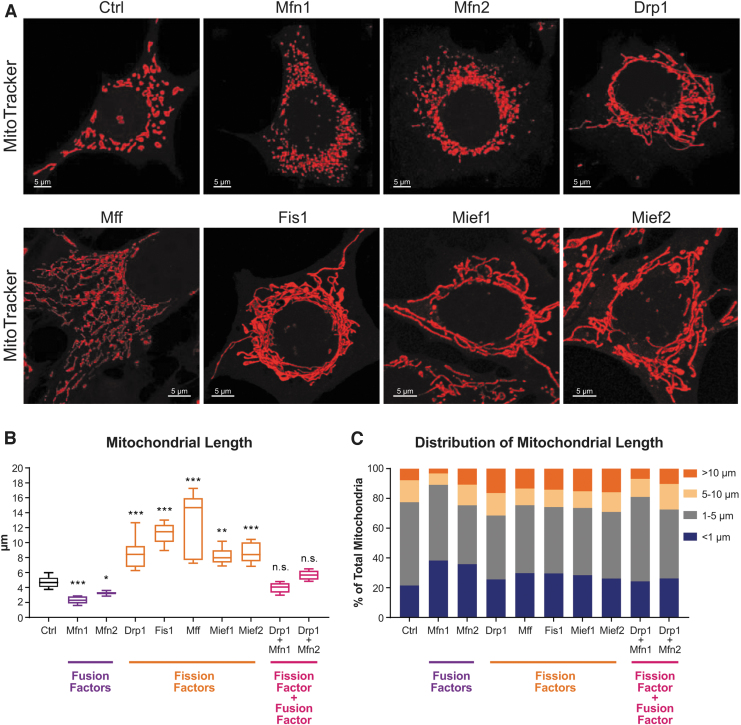
ASOs targeting mitochondrial fission and fusion factors modulate mitochondrial morphology. **(A)** Representative confocal microscopy images of MHT cells treated with 5 μM of indicated ASOs for 48 h and stained with MitoTracker-Red-CMX-Ros to visualize mitochondria. Scale bars are 5 μm. **(B)** Average mitochondrial length (*n* = 6–9 images per group, one-way ANOVA with Dunnett's multiple comparison test, *P < 0.05, **P < 0.005, ***P < 0.0005). Data are box plots from one of two separate experiments, with the center lines representing medians. *Box limits* indicate the 25th and 75th percentiles. *Whiskers* extend to the 5th and 95th percentiles. **(C)** Distribution of mitochondrial lengths as a percentage of total mitochondria quantified from all images in each group.

Given that decreasing the levels of mitochondrial fission and fusion factors has been widely reported to alter mitochondrial network structure, we next examined how ASO treatments change mitochondrial morphology *in vitro*. MHT cells treated with fusion or fission ASOs at 5 μM for 48 h exhibited dramatic alterations in mitochondrial size when visualized with MitoTracker (Fig. 2A). Specifically, ASOs targeting fusion factors *Mfn1* and *Mfn2* decreased mean mitochondrial length, while the opposite was observed with ASOs targeting fission factors *Drp1*, *Fis1*, *Mff*, *Mief1*, and *Mief2* (Fig. 2B). ASO-dependent changes in mitochondrial length distribution were also observed, with fusion factor ASOs increasing the proportion of small mitochondria (<1 μm) and fission factor ASOs increasing the proportion of large mitochondria (>10 μm; Fig. 2C). Interestingly, ASO-dependent changes in mitochondrial size were prevented when MHT cells were treated with two opposing ASOs simultaneously. Coadministration of one fission factor ASO together with one fusion factor ASO led to a normalization of mitochondrial sizes (Fig. 2B, C, and Supplementary Fig. S2D). These data show that modulation of mitochondrial morphology is possible through ASO-mediated gene silencing and that it occurs rapidly within 48 h.

### ASOs targeting mitochondrial fusion and fission factors alter mitochondrial respiration

Since changes in mitochondrial morphology have been linked to changes in function, we next sought to determine if ASO-mediated silencing of mitochondrial fusion and fission factors alters mitochondrial functional parameters. Previous reports have shown a direct association between mitochondrial fragmentation and decreased mitochondrial respiratory function, and fragmentation is often a prerequisite to apoptosis [30,31]. Changes in mitochondrial morphology have also been linked to alterations in mitochondrial membrane potential, a necessary component of oxidative phosphorylation (OXPHOS)-dependent ATP production [32]. Based on these studies, we hypothesized that ASO-mediated elongation of mitochondrial networks would lead to an increase in respiration capacity, while ASO-mediated fragmentation would cause a decrease in respiration.

**FIG. 3.**
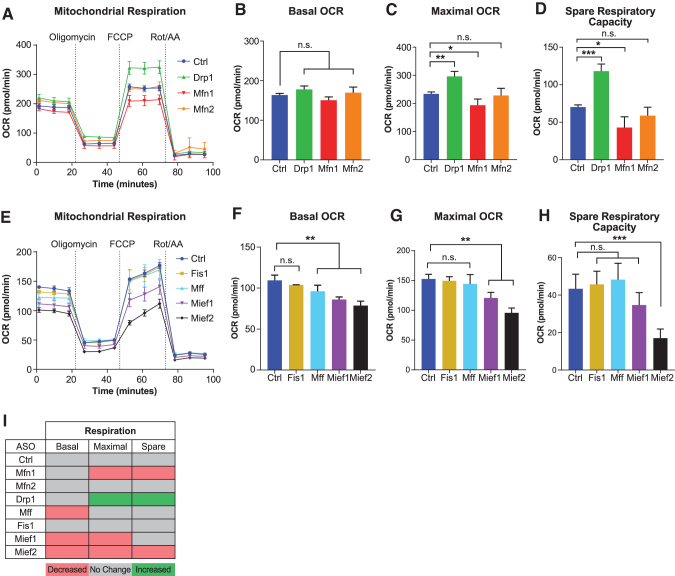
ASOs targeting mitochondrial fission and fusion factors alter mitochondrial respiration. Mitochondrial respiration measured by OCR over time in MHT cells treated with 5 μM of indicated ASOs for 48 h prior **(A, E)**. Assay compounds were added at indicated timepoints (Oligomycin, FCCP, Rot/AA). Basal respiration **(B, F)**, maximal respiration **(C, G)**, and spare capacity **(D, H)** calculated from experiments in **(A, E)**, respectively. **(I)** Summary of data shown in **A–H**. All data shown are from individual experiments representative of at least two separate experiments and reported as mean ± SEM, *n* = 4 **(A–E)**, one-way ANOVA with Dunnett's multiple comparison test, *P < 0.05, **P < 0.005, ***P < 0.0005. FCCP, trifluoromethoxy carbonylcyanide phenylhydrazone. OCR, oxygen consumption rate; Rot/AA, rotenone/antimycin A.

To measure mitochondrial respiration, we treated MHT cells with 5 μM ASOs for 48 h and performed oxygen consumption analysis using the Seahorse XFe24 Analyzer [33]. We found that basal oxygen consumption is largely unchanged upon fusion/fission factor ASO administration, although Mff, Mief1, and Mief2 ASOs did cause a slight decrease (Fig. 3A, B, E, F). Importantly, we found that maximal respiration and spare respiratory capacity were significantly affected by most ASO treatments (Fig. 3C, D, G, H). Surprisingly, however, only the Drp1 ASO enhanced maximal oxygen consumption and spare respiratory capacity, suggesting that not all forms of mitochondrial network elongation affect respiration in the same way and that additional functions of the fission factors may play a role.

Mfn1, Mief1, and Mief2 ASOs caused a decrease in oxygen consumption parameters, suggesting that mitochondrial dysfunction can occur in the context of mitochondrial fragmentation, as well as elongation. Mfn2 ASO did not change oxygen consumption parameters, despite causing mitochondrial fragmentation, suggesting that MFN2 is dispensable for mitochondrial function in MHT cells. Together, these data show that mitochondrial morphology does not always correlate with mitochondrial function and that one strategy to enhance function is by Drp1 ASO-mediated mitochondrial elongation (Fig. 3I).

### ASOs targeting mitochondrial fusion and fission factors alter total mitochondrial content

**FIG. 4.**
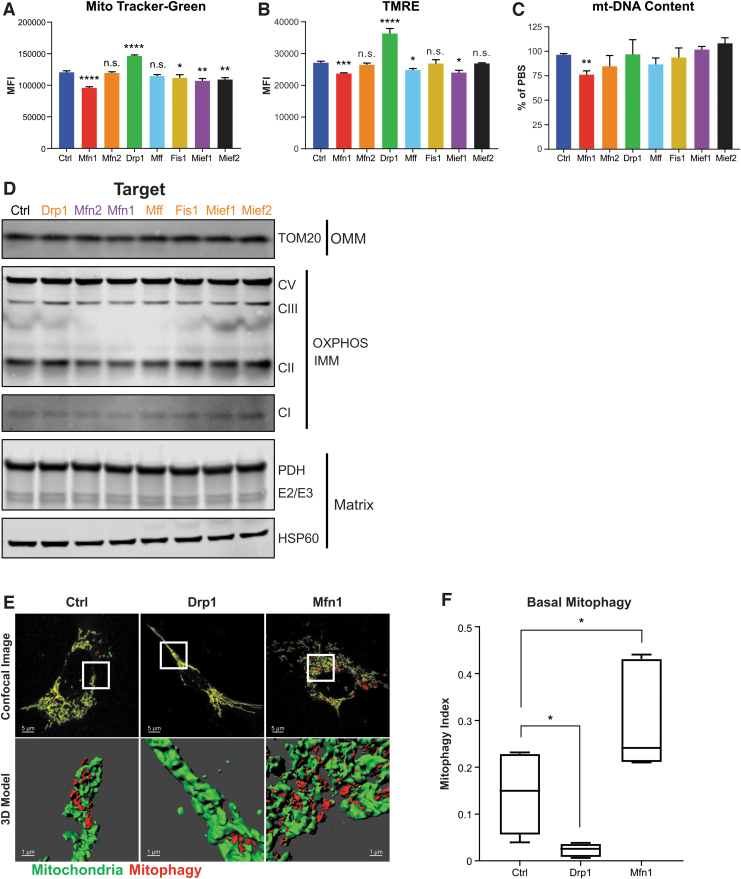
ASOs targeting mitochondrial fission and fusion factors modulate mitochondrial content and basal mitophagy. Total mitochondrial mass **(A)** and mitochondrial membrane potential **(B)** quantified by flow cytometry of MHT cells treated with 5 μM of indicated ASOs for 48 h, then stained with the indicated fluorescent dyes (MitoTracker-Green, TMRE). **(C)** Mitochondrial DNA content of MHT cells treated with indicated ASOs quantified as the ratio of mitochondrial DNA gene (*mt-Nd2*) to nuclear DNA gene (*Gusb*) and normalized to cells treated with ASO vehicle (% of PBS). **(D)** Western blots of whole cell lysates of MHT cells treated with 5 μM of indicated ASOs for 48 h. Primary antibodies used to detect protein levels are indicated. **(E)** Representative confocal microscopy images and corresponding 3D reconstructions of MHT cells stably expressing the mitophagy reporter Mito-QC and treated with 5 μM of indicated ASOs for 48 h before imaging. Scale bars are 5 μm for the *upper panels* and 1 μm for the *lower panels*. **(F)** Mitophagy was quantified by calculating the Mitophagy Index, defined as the ratio of the total volume of mitophagy events to the total volume of mitochondria per cell (see Materials and Methods section for details). Data are box plots from one of two separate experiments, with the center lines representing medians. *Box limits* indicate the 25th and 75th percentiles. *Whiskers* extend to the 5th and 95th percentiles (*n* = 6–9 images per group, one-way ANOVA with Dunnett's multiple comparison test, *P < 0.05). All other data are shown as mean ± SEM, *n* = 3, one-way ANOVA with Dunnett's multiple comparison test, *P < 0.05, **P < 0.005, ***P < 0.0005, ****P < 0.00005. CI, CII, CIII, CV, OXPHOS complex subunits; 3D, three-dimensional; IMM, inner mitochondrial membrane; MFI, mean fluorescence intensity; OMM, outer mitochondrial membrane; OXPHOS, oxidative phosphorylation; TMRE, tetramethylrhodamine ethyl ester.

To determine if ASO-dependent changes in mitochondrial function were caused by changes in total mitochondrial content or membrane potential, we used flow cytometry to quantify total mitochondrial mass (MitoTracker Green) and mitochondrial membrane potential (TMRE) in MHT cells treated with 5 μM ASOs for 48 h. Interestingly, of all the fission factor ASOs, only the Drp1 ASO caused a significant increase in total mitochondrial mass (Fig. 4A), despite all causing mitochondrial network elongation (Fig. 2). In contrast, Mief1 and Mief2 ASOs caused a decrease in mitochondrial mass (Fig. 4A) despite a clear increase in mitochondrial length (Fig. 2), in agreement with their detrimental effects on mitochondrial respiration (Fig. 3E).

When we evaluated fusion factor ASOs, we found that Mfn1 ASO caused a decrease in total mitochondrial mass, while it remained unchanged with Mfn2 ASO treatment (Fig. 4A), despite both causing mitochondrial fragmentation (Fig. 2). This observation is in agreement with the disparate effects of Mfn1 and Mfn2 ASO treatment on mitochondrial respiration (Fig. 3A). Drp1 ASO also caused an increase in membrane potential, while Mfn1 ASO caused a decrease (Fig. 4B), although these changes are likely due to corresponding changes in total mitochondrial mass (Fig. 4A).

mt-DNA content, another readout for total mitochondrial mass, was largely unchanged across ASO treatments, except for Mfn1 ASO which caused a decrease (Fig. 4C). We found similarly, by western blot of mitochondrial markers, that proteins from the various mitochondrial compartments (outer mitochondrial membrane, OMM; inner mitochondrial membrane, IMM; matrix) are largely unchanged for most ASOs (Fig. 4D and Supplementary Fig. S3). However, we found that Drp1 ASO increased, while Mfn1 ASO decreased, levels of the OXPHOS components Complex II (CII) and Complex III (CIII), consistent with their effects on other readouts of total mitochondrial content and respiration (Figs. 3A and 4A–C). Overall, these data suggest that ASO-mediated changes in mitochondrial morphology may be linked to changes in function by their effects on mitochondrial content and mitochondrial membrane potential. However, it remains unclear why ASO-mediated mitochondrial elongation results in divergent effects on mitochondrial mass and function. Moreover, these data underscore the importance of utilizing multiple methods to determine total mitochondrial content in cells.

### ASOs targeting mitochondrial fusion and fission factors alter basal mitophagy

Previous studies have shown that mitochondrial dynamics plays a role in modulating levels of mitophagy, which has effects on cellular mitochondrial content [34,35]. Mitophagy is the major intracellular pathway of mitochondrial removal and is known to influence mitochondrial functions [36–38]. During mitophagy, adaptor proteins P62/SQSTM and OPTN accumulate on the outer membrane of damaged mitochondria and recruit autophagosomes by their interactions with LC3B [39]. LC3B is an autophagosome membrane component whose lipidated form (LC3B-II) is a common readout for autophagy pathway activation [40]. Given our results suggesting differential effects of mitochondrial fusion and fission factor ASOs on mitochondrial function and total mitochondrial content, we next sought to determine if basal mitophagy is altered by these ASOs.

To investigate ASO-mediated effects on mitophagy, we focused on Drp1 and Mfn1 ASOs due to their clear effects on mitochondrial morphology, function, and content in MHT cells (Figs. 2 and 3). First, we evaluated the effects of Drp1 and Mfn1 ASOs on autophagy and mitophagy by western blot analysis and found that 5 μM treatment of MHT cells for 48 h did not cause obvious changes in LC3B, P62, and OPTN levels (Supplementary Fig. S4). These results suggest that global changes in autophagy and mitophagy are not being induced by ASO-mediated silencing of *Drp1* or *Mfn1*. However, Drp1 ASO treatment did cause a slight increase in P62, suggesting that there could be an accumulation of mitophagy intermediates indicative of a delay in mitophagy, as previously reported [41,42].

To monitor basal mitophagy events in a more sensitive and quantitative manner, we used MHT cells stably expressing the lentiviral mitophagy reporter, Mito-QC [29,43]. Mito-QC is an outer mitochondrial membrane-targeted, pH-sensitive, tandem fluorescent reporter (mCherry-GFP). In the low pH environment of lysosomes, GFP fluorescence is quenched, and the remaining mCherry-only signals represent mitophagic events (mitochondrial fragments within lysosomes). Three-dimensional modeling of confocal images provides a quantitative readout of basal mitophagy levels. Using MHT cells stably expressing Mito-QC, we found that basal mitophagy is altered by ASO-mediated reduction of DRP1 and MFN1 (5 μM, free uptake for 48 h). Drp1 ASO treatment decreased, while Mfn1 ASO treatment increased, the level of basal mitophagy (Fig. 4E, F) suggesting that Drp1 and Mfn1 ASO treatment may be affecting mitochondrial content and, in turn, the function by modulating basal mitophagy.

Together, these results show that ASO-dependent modulation of mitochondrial morphology influences basal mitophagy in the absence of overt mitochondrial damage or stress. In addition, these results provide a mechanism that links the ASO-mediated changes in mitochondrial content with mitochondrial function, suggesting that effects on total mitochondrial content are likely due to modulation of the pathway that orchestrates their removal and degradation. Moreover, these results support the notion that mitochondrial fission is a necessary step in mitophagy, as previously reported [34], and that enabling excess mitochondrial fusion (by inhibiting fission) is sufficient to restrict basal mitophagic events. Conversely, enabling excess mitochondrial fission (by inhibiting fusion) is sufficient to enhance basal mitophagy, suggesting that mitochondrial size is an important factor in autophagosome engulfment during mitophagy.

### Changes in mitochondrial morphology and respiration are ASO-dose dependent

**FIG. 5.**
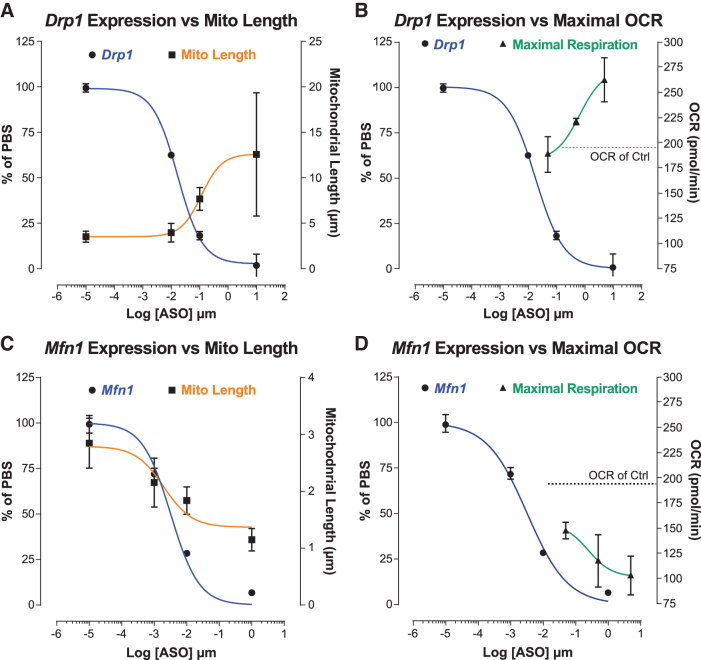
Mitochondrial length and mitochondrial respiration are ASO dose dependent. *Drp1* expression versus mitochondrial length **(A)** and *Drp1* expression versus maximal OCR **(B)** in MHT cells treated with indicated Drp1 ASO concentrations for 48 h. *Mfn1* expression versus mitochondrial length **(C)** and *Mfn1* expression versus maximal OCR **(D)** in MHT cells treated with indicated Mfn1 ASO concentrations for 48 h. Gene expression, mitochondrial length, and maximal OCR were measured in separate parallel experiments. Dose–response curve fits for gene expression, mitochondrial length, and maximal OCR were determined using nonlinear regressions with least-squares fit in Prism (GraphPad). *Horizontal dotted lines* represent the mean OCR in cells treated with 5 μM Control ASO.

To evaluate the dose-responsive effects of Drp1 and Mfn1 ASOs on mitochondrial morphology, we treated MHT cells with Drp1 and Mfn1 ASOs in separate dose–response experiments, then analyzed target gene expression, and quantified mitochondrial morphology. As observed previously, Drp1 and Mfn1 ASO treatments resulted in dose responsive reductions in *Drp1* and *Mfn1* expression (Fig. 5A, C). Similarly, we found that both Drp1 and Mfn1 ASOs also modulated mitochondrial length in a dose-dependent manner (Fig. 5A, C). Interestingly, we found significant differences in the sensitivity of MHT cells to ASO-mediated reductions in *Drp1* compared to *Mfn1*. We observed that at least 50%–80% reduction of *Drp1* expression is necessary to observe an increase in mitochondrial length, while a minimum of only 25%–40% reduction of *Mfn1* expression is needed to observe a decrease in mitochondrial length (Fig. 5A, C).

We found similar ASO dose-dependent effects on mitochondrial function. In three-point ASO dose–response experiments, a dose-dependent increase in maximal respiration (OCR) was observed for Drp1 ASO, while the opposite was observed for Mfn1 ASO (Fig. 5B, D). Reflecting the ASO dose-dependent effects on mitochondrial morphology, enhancement of maximal respiration required more than ∼80% reduction of *Drp1*, while a decrease in maximal respiration was observed at all Mfn1 ASO concentrations tested (>80% *Mfn1* reduction). All together, these data suggest that for Drp1 and Mfn1 ASOs the effects on mitochondrial morphology and function are likely linked and can be modulated incrementally in response to ASO dose. Furthermore, these data showcase the enhanced functional sensitivity of cells to mitochondrial fragmentation—as opposed to mitochondrial elongation—shedding light on the dose requirements needed to effectively modulate mitochondrial dynamics in cells with ASOs.

### ASO-mediated silencing of Drp1, but not other fission factors, rescues mitochondrial fragmentation and dysfunction in *Mfn1* KO and MFN2-R94Q MEFs

To test whether ASO-mediated modulation of mitochondrial dynamics can rescue mitochondrial dysfunction in cells with perturbed mitochondrial morphology, we utilized two MFN KO MEF cell lines as *in vitro* model systems of excess mitochondrial fragmentation: (1) *Mfn1* KO MEFs and (2) *Mfn2* KO MEFs stably expressing exogenous *Mfn2* harboring a CMT2A mutation R94Q [44], referred to from now on as MFN2-R94Q. Both cell lines exhibit mitochondrial fragmentation and dysfunction compared to WT MEFs (Supplementary Fig. S5A), as reported previously [30,45,46]. MFN2-R94Q expression was confirmed in lentivirally transduced *Mfn2* KO MEFs at the protein and mRNA level (Supplementary Fig. S5B). We sought to correct the mitochondrial dysfunction in these cells with treatment of fission factor ASOs in an attempt to restore mitochondrial homeostasis. We hypothesized that despite the negative effects on respiration of some fission factor ASOs in unperturbed cells (Fig. 3E–I), treatment of fission factor ASOs in a disease context may provide a beneficial effect.

**FIG. 6.**
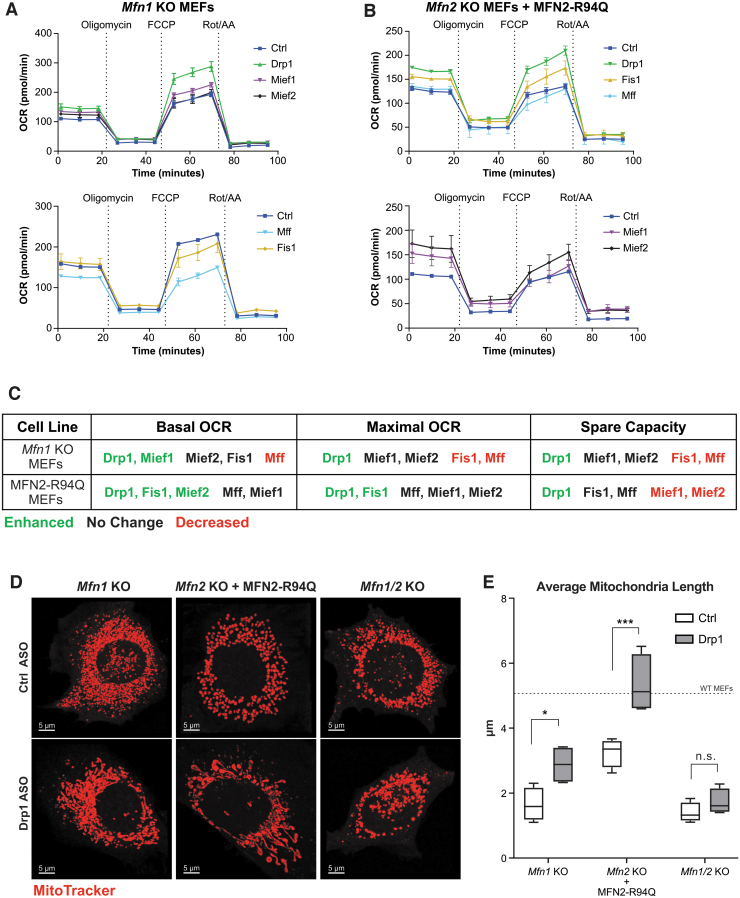
ASO-mediated silencing of Drp1 rescues mitochondrial dysfunction in *Mfn1* KO and MFN2-R94Q MEFs. Mitochondrial respiration measured by OCR over time in *Mfn1* KO MEFs **(A)** and MFN2-R94Q MEFs **(B)** treated with the 5 μM of indicated ASOs 48 h prior. Assay compounds were added at indicated timepoints. Data shown are from individual experiments representative of at least two separate experiments and reported as mean ± SEM, *n* = 4. See Supplementary Fig. 5 for basal OCR, maximal OCR, and spare respiratory capacity measures. **(C)** Table summarizing the effects of indicated ASOs on mitochondrial respiration in *Mfn1* KO and MFN2-R94Q MEFs. **(D)** Representative confocal microscopy images of *Mfn1* KO, MFN2-R94Q, and *Mfn1/2* KO MEFs treated with 5 μM of indicated ASOs for 48 h and stained with MitoTracker-CMX-Ros to visualize mitochondria. Scale bars are 5 μm. **(E)** Average mitochondrial length (*n* = 4 images per group, two-way ANOVA with Sidak's multiple comparisons test, *P < 0.05, ***P < 0.0005). Data are box plots from one of two separate experiments, with the center lines representing medians. *Box limits* indicate the 25th and 75th percentiles. *Whiskers* extend to the 5th and 95th percentiles. KO, knockout; MEFs, mouse embryonic fibroblasts.

To measure the effects of ASOs on mitochondrial respiration in MEFs, we performed oxygen consumption analysis using the Seahorse XFe24 Analyzer. Administration of fission factor ASOs to MEF cell lines by free uptake at 5 μM for 48 h caused significant changes in mitochondrial function (Fig. 6A, B). In *Mfn1* KO MEFs, Drp1 ASO enhanced basal OCR, maximal OCR, and spare respiratory capacity, while ASOs targeting other fission factors were either detrimental to or did not change respiration (Fig. 6A and Supplementary Fig. S5C, F). Similarly, Drp1 ASO consistently enhanced basal OCR, maximal OCR, and spare respiratory capacity in MFN2-R94Q MEFs (Fig. 6B and Supplementary Fig. S5C, G–I). However, unlike in *Mfn1* KO MEFs, Fis1 ASO enhanced basal and maximal OCR, but did not spare respiratory capacity (Supplementary Fig. S5G–I). Although Mief1 and Mief2 ASOs decreased spare capacity, other ASOs targeting fission factors did not greatly change mitochondrial function in MFN2-R94Q MEFs (Fig. 6B and Supplementary Fig. S5G–I). Paradoxically, Mief2 ASO caused an increase in basal OCR, in the absence of an increase in other OCR measures (Supplementary Fig. S5G–I). Overall, only Drp1 ASO enhanced the various OCR measures in both cellular models of mitochondrial dysfunction (Fig. 6C).

Since Drp1 ASO was the only fission factor ASO that consistently enhanced mitochondrial function in *Mfn1* KO and MFN2-R94Q MEFs, we next sought to evaluate the effects of Drp1 ASO treatment on mitochondrial morphology in these cells. In accordance with enhanced mitochondrial function, Drp1 ASO caused an increase in mitochondrial length in both *Mfn1* KO and MFN2-R94Q MEFs (Fig. 6D, E). Interestingly, Drp1 ASO failed to restore mitochondrial morphology in *Mfn1/Mfn2* double KO MEFs, suggesting that the presence of either MFN1 or MFN2 is necessary for Drp1 ASO-mediated rescue.

Together, these results provide a novel proof-of-concept for ASO-mediated rescue of mitochondrial morphology and function in contexts of mitochondrial dysfunction due to an imbalance in mitochondrial dynamics. Furthermore, the data suggest that while Drp1 ASO consistently rescued mitochondrial dysfunction, the functional effects of targeting *Mff*, *Fis1*, *Mief1*, and *Mief2* are likely context dependent. Notably, Drp1 ASO was able to overcome the dominant negative nature of the R94Q mutation in MFN2 suggesting that other dominant negative mutations affecting mitochondrial fusion may be amenable to this approach.

## Discussion

The dynamic regulation of mitochondrial shape is essential for the various roles mitochondria play in the cell. Metabolic adaptation, stress response, apoptosis, and even oogenesis and fertilization require mitochondrial dynamism through the action of mitochondrial fission and fusion factors [47–49]. With the growing number of diseases that are associated with mitochondrial dysfunction [50], it is critical to discover novel therapeutics that aim to restore mitochondrial homeostasis.

This study reports, for the first time, the use of ASOs to systematically evaluate the effects of reducing fission and fusion factors on both mitochondrial morphology and mitochondrial respiration in unperturbed cells in a dose-dependent manner. Furthermore, we demonstrate that a *Drp1*-targeting ASO, but not other fission factor ASOs, can rescue mitochondrial dysfunction in two cellular disease models, including one containing a disease-relevant dominant-negative mutation in MFN2 (R94Q). Altogether, this study is a proof-of-concept for the beneficial rebalancing of mitochondrial morphology to restore mitochondrial function and paves the way for further drug discovery for various mitochondrial genetic diseases for which there is significant unmet medical need.

Targeting mitochondrial fission pharmacologically has been previously reported using peptides and small molecules in various disease models with some success. The peptide P110 inhibits DRP1's association with FIS1 and caused increased mitochondrial size and membrane potential, in agreement with the present study [51]. Importantly, P110 prevented neurotoxicity in multiple mouse models and prevented *Escherichia coli*-mediated mitochondrial fragmentation associated with gut permeability in Crohn's disease [52–54]. Mdivi-1, a small molecule inhibitor of DRP1's GTPase activity, has also been used to prevent disease-associated mitochondrial fragmentation; however, more recently the specificity and mechanism of action of Mdivi-1 have come into question [55].

In contrast, small-molecule peptide mimics have been used to restore mitochondrial fusion activity in disease models *in vitro* through allosteric activation of MFN1 and MFN2 [56]. While these efforts represent significant advancements in the field, more studies are needed to fully characterize the specificity and off-target effects of these compounds before their advancement into the clinic. ASOs, on the other hand, present a more targeted approach to modulating mitochondrial fission and fusion and possess a clinically favorable profile [57,58].

In this study, we show how reducing DRP1 with ASOs enhances mitochondrial function in both unperturbed cells and models of mitochondrial dysfunction. We attribute this effect to the reduction of mitochondrial removal through the mitophagy pathway, as similarly described in previous studies [34,48,59]. However, the functional effects of reducing DRP1 in cells are mixed in the literature. For instance, one study reported reduced mitochondrial respiration in cardiomyocytes lacking *Drp1* [42], while another reported enhanced respiration in *Drp1* KO MEFs [60]. These discrepancies support the notion that the effect of DRP1 reduction and mitophagy is cell-type specific. While long-term developmental reduction of DRP1 is likely detrimental to unperturbed cells due to reductions in mitophagy, we show here that a transient reduction in DRP1 using ASOs may be a safer approach to restoring mitochondrial homeostasis.

It has not escaped our notice that there are discrepancies in the mitochondrial morphology-function relationship outside of our focus on DRP1 and MFN1. We found that ASO-mediated reduction of MFN2 did not alter mitochondrial function while MFN1 did, suggesting that MHT cells don't require MFN2 to sustain proper respiration in the face of mitochondrial fragmentation. Differential functions of MFN1 and MFN2 have been reported previously suggesting a potential reason for this finding [61]. In addition, the increase in mitochondrial length that we reported with ASOs targeting *Mff*, *Fis1*, *Mief1*, and *Mief2* and the resulting lack of mitochondrial mass and respiration enhancement is counterintuitive.

It is possible that DRP1's extramitochondrial effects may be influencing respiration in this context. For instance, DRP1 is a known regulator of peroxisome fission [62,63], and we observed elongated peroxisomes in MHT cells treated with Drp1 ASO (data not shown). Furthermore, peroxisomes perform beta-oxidation of fatty acids, and we found that MHT cells treated with Drp1 ASO have an enhanced dependence on fatty acids as fuel for mitochondrial respiration (data not shown). This shift in fuel preference, in combination with increased mitochondrial content, may be contributing to the enhancement in respiration observed with Drp1 ASO.

In contrast, it is possible that additional known or unknown functions of MFF, FIS1, MIEF1, and MIEF2, other than their roles in mitochondrial fission, may counteract the beneficial effects of suppressed fission on mitochondrial respiration. These are interesting hypotheses that are beyond the scope of this study and may be the focus of future studies. Nevertheless, our systematic evaluation of ASOs targeting each outer mitochondrial membrane fission factor is the first study to show the effects each fission factor has on mitochondrial morphology and function in unperturbed cells, as well as effects on respiration in an *in vitro* disease model of CMT2A.

The use of ASOs provides the benefit of being able to fine-tune mRNA expression level in a dose-dependent manner, which we show leads to concomitant changes in mitochondrial morphology and function. While previous studies have shown that a partial reduction in *Drp1* expression results in significant changes in mitochondrial morphology [64], our study is the first to our knowledge to explore this notion further in multidose–response experiments. These findings have important implications for ASO drug development since knowing how much target reduction is required to observe a functional effect (eg, mitochondrial respiration) can relieve potential on-target liabilities in off-target tissues. Overall, this study provides novel proof-of-concept for the use of ASOs in restoring the balance of mitochondrial homeostasis in the context of disease and paves the way for future ASO drug development for the increasing number of diseases associated with mitochondrial dysfunction.

## Supplementary Material

Supplemental data

Supplemental data

Supplemental data

Supplemental data

Supplemental data

Supplemental data
