# Supplementary material for: Antisense Oligonucleotide-Mediated Silencing of Mitochondrial Fusion and Fission Factors Modulates Mitochondrial Dynamics and Rescues Mitochondrial Dysfunction
Source: Nucleic Acid Ther. 2022 Jan 31;32(1):51–65. doi: 10.1089/nat.2021.0029 (PMC8817704; doi:10.1089/nat.2021.0029)
Supplement: Supplemental data [file Supp_DataS1.docx]

**Supplementary Materials**

Primary Antibodies

| **Target** | **Source** | **Catalog number** |
| --- | --- | --- |
| DRP1 | BD Bioscience | 611112 |
| MFN1 | Novus Biologicals | NBP1-71775 |
| MFN2 | Abcam | ab56889 |
| MFF | Cell Signaling | 86668 |
| FIS1 | Proteintech | 10956-1-AP |
| MIEF1 | Proteintech | 20164-1-AP |
| MIEF2 | Santa Cruz Biotechnology | sc-515800 |
| TOM20 | Proteintech | 11802-1-AP |
| ACTB | Thermo Fisher Scientific | MA5-15739 |
| LC3B | Novus Biologicals | NB100-2220 |
| P62 | Progen | GP62-C |
| OPTN | Proteintech | 10837-1-AP |
| HSP60 | Thermo Fisher Scientific | MA3-012 |

Secondary Antibodies

| **Secondary Antibodies** | **Source** | **Catalog number** |
| --- | --- | --- |
| IRDye® 800CW Goat anti-Rabbit IgG | LI-COR | 926-32211 |
| IRDye® 800CW Goat anti-Mouse IgG | LI-COR | 926-32210 |
| IRDye® 800CW Donkey anti-Guinea Pig IgG | LI-COR | 926-32411 |
| IRDye® 680RD Goat anti-Rabbit IgG | LI-COR | 926-68071 |
| IRDye® 680RD Goat anti-Mouse IgG | LI-COR | 926-68070 |
| IRDye® 680RD Donkey anti-Guinea Pig IgG | LI-COR | 926-68077 |

Primer and Probe Sequences

| **Target** | **Species** | **Forward Primer (5'-3')** | **Reverse Primer (5'-3')** | **Probe (5'-3')** |
| --- | --- | --- | --- | --- |
| *Drp1* | *M. Musculus* | CCCTAAACTTCACGATGCCA | GCTCAATTGCCACTAAGTTATGC | TGTCTTCTTCGTAAAAGGTTGCCCGT |
| *Mfn1* | *M. Musculus* | AGCCATCACTGCAATCTTCG | TTCTGTAGCCCTGTATTTCCAC | TGTGAGCCCTCAGTAACAAACTCCAG |
| *Mfn2* | *M. Musculus* | GATGGACTATGCTAGTGAACAGG | GTTGGCAGGTGTCAGAGG | CTAGGCTACAGTGATCAGGTTCAGCG |
| *Mff* | *M. Musculus* | GCAGCTTCATTAAGACGTCAGATAA | GAAACCAGAGCCAGCTGTTAAG | TCTGGAAGAGGAGAATAAAGAGCGTGC |
| *Fis1* | *M. Musculus* | CGAAGCAAATACAATGAGGACATC | ACGGCCAGGTAGAAGACATAG | CAAAGGGAGCAAAGAGGAACAGCG |
| *Mief1* | *M. Musculus* | GGACATGTACTTGAGTGGCA | GGGACAATGAGTTGGATATGGT | TGATGATCTGCAGGTGGTGACGG |
| *Mief2* | *M. Musculus* | CTACTCGCCCTGTCCTTG | CCAATCGAGCATTAGCCAAGA | TCGGACGCAGGTGACCATGG |
| *Ppia (Cyclophilin A)* | *M. Musculus* | TCGCCGCTTGCTGCA | ATCGGCCGTGATGTCGA | CCATGGTCAACCCCACCGTGTTC |
| *nuc-Gusb* | *M. Musculus* | Taqman Assay from Thermo Fisher Scientific (Mm03003537_s1) |  |  |
| *mt-Nd2* | *M. Musculus* | Taqman Assay from Thermo Fisher Scientific (Mm04225288_s1) |  |  |

Antisense Oligonucleotide Sequences

| **Oligo Name** | **Target** | **Species** | **Length** | **Sequence (5’-3’)** |
| --- | --- | --- | --- | --- |
| Control (Ctrl) ASO | n/a | n/a | 16 | GGCCAATACGCCGTCA |
| Mfn1 ASO | *Mfn1* | *M. musculus* | 16 | AGAGTATAGTGTAGTT |
| Mfn2 ASO | *Mfn2* | *M. musculus* | 16 | GTATATGCATCTGAGC |
| Drp1 ASO | *Drp1* | *M. musculus* | 16 | GGTAATAAGCTGGAGT |
| Mff ASO | *Mff* | *M. musculus* | 16 | GACAATTTTTGACCAT |
| Fis1 ASO | *Fis1* | *M. musculus* | 16 | ACACTATATTAACGGC |
| Mief1 ASO | *Mief1* | *M. musculus* | 16 | TCAATTAAAGCTTCAG |
| Mief2 ASO | *Mief2* | *M. musculus* | 16 | CCTCATTTACCTAGAA |

n/a: not applicable

Underlining indicates constrained ethyl (cEt)-modified bases
