# Supplementary material for: Antisense Oligonucleotide-Mediated Silencing of Mitochondrial Fusion and Fission Factors Modulates Mitochondrial Dynamics and Rescues Mitochondrial Dysfunction
Source: Nucleic Acid Ther. 2022 Jan 31;32(1):51–65. doi: 10.1089/nat.2021.0029 (PMC8817704; doi:10.1089/nat.2021.0029)
Supplement: Supplemental data [file Supp_FigS2.docx]

**Supplementary Figure 2. Gene expression of mitochondrial fission and fusion factors is not altered after ASO administration.** Mitochondrial fusion factor (A) and fission factor (B) gene expression measured by qPCR in MHT cells treated with 5 μM of indicated ASOs. (C) Quantification of western blots shown in Fig. 1C. Relative band density was determined using ImageJ. Relative density for each band was normalized to the relative density of ACTB for each respective sample. (D) Gene expression measured by qPCR in MHT cells after 48 hr of co-administration of two different ASOs.
