# Supplementary material for: Antisense Oligonucleotide-Mediated Silencing of Mitochondrial Fusion and Fission Factors Modulates Mitochondrial Dynamics and Rescues Mitochondrial Dysfunction
Source: Nucleic Acid Ther. 2022 Jan 31;32(1):51–65. doi: 10.1089/nat.2021.0029 (PMC8817704; doi:10.1089/nat.2021.0029)
Supplement: Supplemental data [file Supp_FigS3.docx]

**Supplementary Figure 3.** Quantification of western blots shown in Fig. 4D. Relative band density was determined using ImageJ.
