# Supplementary material for: Antisense Oligonucleotide-Mediated Silencing of Mitochondrial Fusion and Fission Factors Modulates Mitochondrial Dynamics and Rescues Mitochondrial Dysfunction
Source: Nucleic Acid Ther. 2022 Jan 31;32(1):51–65. doi: 10.1089/nat.2021.0029 (PMC8817704; doi:10.1089/nat.2021.0029)
Supplement: Supplemental data [file Supp_FigS4.docx]

**Supplementary Figure 4.** Representative western blot images of MHT whole cell lysates treated with 5 μM of indicated ASOs for 48 hours prior to lysis. Primary antibodies used to detect autophagy and mitophagy protein levels are indicated. Beta-Actin (ACTB) served as a loading control. Vertical spaces inserted between lanes indicate removal of intervening, irrelevant samples. All samples were run on the same gel, transferred and blotted together, and imaged in a single scan.
