# Supplementary material for: Antisense Oligonucleotide-Mediated Silencing of Mitochondrial Fusion and Fission Factors Modulates Mitochondrial Dynamics and Rescues Mitochondrial Dysfunction
Source: Nucleic Acid Ther. 2022 Jan 31;32(1):51–65. doi: 10.1089/nat.2021.0029 (PMC8817704; doi:10.1089/nat.2021.0029)
Supplement: Supplemental data [file Supp_FigS5.docx]

**Supplementary Figure 5. ASO-mediated silencing of Drp1 rescues mitochondrial dysfunction in *Mfn1* KO and MFN2-R94Q MEFs.** (A) Mitochondrial respiration measured by oxygen consumption rate (OCR) over time in *Mfn1* KO MEFs (left) and MFN2-R94Q MEFs (right) compared to WT MEFs. (B) Western blot of whole cell lysates (left) and qPCR (right) showing that stable expression of MFN2-R94Q lentiviral vector was achieved in *Mfn2* KO MEFs. (C) Western blot of whole cell lysates showing 80% reduction in DRP1 protein level in *Mfn1* KO and MFN2-R94Q MEFs treated with Drp1 ASO compared to Control ASO (5 μM, 48 hours). (D-F) Basal OCR, maximal OCR, and spare respiratory capacity measures from data shown in Figure 6A. (G-I) Basal OCR, maximal OCR, and spare respiratory capacity measures from data shown in Figure 6B. All data in A-I are shown as means ± SEM, *n* = 4, one-way ANOVA with Dunnett’s multiple comparison test, * *P* < 0.05, ** *P* < 0.005, *** *P* < 0.0005.
